# Localizing Perturbations in Pressurized Water Reactors Using One-Dimensional Deep Convolutional Neural Networks

**DOI:** 10.3390/s22010113

**Published:** 2021-12-24

**Authors:** Laurent Pantera, Petr Stulík, Antoni Vidal-Ferràndiz, Amanda Carreño, Damián Ginestar, George Ioannou, Thanos Tasakos, Georgios Alexandridis, Andreas Stafylopatis

**Affiliations:** 1 CEA, DES, IRESNE, DER, SPESI, LP2E, Cadarache, 13108 Saint-Paul-Lez-Durance, France; laurent.pantera@cea.fr; 2 Nuclear Research Institute, 250 68 Řež, Czech Republic; petr.stulik@ujv.cz; 3 Instituto de Seguridad Industrial, Radifísica y Medioambiental Universitat Politècnica de València, Camino de Vera s/n, 46022 Valencia, Spain; anvifer2@upv.es (A.V.-F.); amcarsan@iqn.upv.es (A.C.); 4 Instituto Universitario de Matemática Multidisciplinar, Universitat Politècnica de València, Camino de Vera s/n, 46022 Valencia, Spain; dginesta@mat.upv.es; 5 Institute of Communication and Computer Systems, Zografou Campus, National Technical University of Athens, 15780 Zografou, Greece; geoioannou@islab.ntua.gr (G.I.); thanostas@islab.ntua.gr (T.T.); andreas@cs.ntua.gr (A.S.)

**Keywords:** neutron noise, neutron diffusion, deep learning, convolutional neural networks, pressurized water reactor, perturbation localization, VVER-1000, absorber of variable strength, FEMFFUSION

## Abstract

This work outlines an approach for localizing anomalies in nuclear reactor cores during their steady state operation, employing deep, one-dimensional, convolutional neural networks. Anomalies are characterized by the application of perturbation diagnostic techniques, based on the analysis of the so-called “neutron-noise” signals: that is, fluctuations of the neutron flux around the mean value observed in a steady-state power level. The proposed methodology is comprised of three steps: initially, certain reactor core perturbations scenarios are simulated in software, creating the respective perturbation datasets, which are specific to a given reactor geometry; then, the said datasets are used to train deep learning models that learn to identify and locate the given perturbations within the nuclear reactor core; lastly, the models are tested on actual plant measurements. The overall methodology is validated on hexagonal, pre-Konvoi, pressurized water, and VVER-1000 type nuclear reactors. The simulated data are generated by the FEMFFUSION code, which is extended in order to deal with the hexagonal geometry in the time and frequency domains. The examined perturbations are absorbers of variable strength, and the trained models are tested on actual plant data acquired by the in-core detectors of the Temelín VVER-1000 Power Plant in the Czech Republic. The whole approach is realized in the framework of Euratom’s CORTEX project.

## 1. Introduction

Nuclear power plants (NPPs) are equipped with many sensors that provide data for the assurance of safety and plant operations, capturing the neutron flux within the core. When the plant is operating under normal conditions, these sensors report steady-state values. In addition to the static component, small fluctuations of the signal may appear, due to inherent fluctuations in the process, caused by a multitude of factors (mechanical vibrations of the fuel assemblies or the core barrel, disturbances in heat transfer fluid flow rates, temperature or density variations, etc). In this sense, an important parameter to observe is the so-called “neutron noise”: that is, the fluctuation of the neutron flux around a mean value, observed in steady-state operating conditions. These fluctuations are important, as they may convey information related to in/out of the core phenomena that can occur as a consequence of initiators (such as temperature or density changes, displacements of core components, etc.), which, in turn, induce fluctuations in neutron cross sections.

The aforementioned phenomena can be grouped in different scenarios [1], such as generic absorbers of variable strength, axially traveling perturbations at the velocity of the coolant flow (e.g., due to fluctuations of the coolant temperature at the inlet of the core), fuel assembly vibrations, control rod vibrations, core barrel vibrations and many others. Those scenarios can be subsequently simulated with computer codes. In this work, perturbation analysis proceeds in two phases. Initially, every perturbation is propagated in the frequency domain across the whole reactor core volume; this is known as the *forward* problem. Thus, there is a one-to-one relationship between every possible location where a perturbation is located and the position where the neutron noise is measured. In operating NPPs, however, the location of the perturbation has to be inferred from measurements originating from neutron detectors (present at specific locations within and out of the core); this is the *backward* problem, for which we have to be able to invert the reactor transfer function. Since inverting the nuclear transfer function is a non-trivial problem, we employ machine and deep learning techniques to this end. The intuition behind this research direction was driven by the authors’ participation in Euratom’s CORTEX project (core monitoring techniques and experimental validation and demonstration) [2], whose objective was to assess the feasibility of methods for exploiting neutron noise in nuclear reactors by employing machine learning models for the inversion of the transfer function and validating the techniques on simulated datasets.

The rest of this paper is organized as follows. In Section 2, we review related work on this subject, while in Section 3, we present the plant and the neutron detector measurements. In Section 4, we give some information about the FEMMFUSION code extensions introduced to take into account the hexagonal geometry and the description of the generated simulated data. In Section 5, we describe the employed deep learning model that locates the perturbation sources distributed in the core. In Section 6, we discuss the performance of the trained model on real plant data, which exhibits promising localization results, when the perturbation occurs around the frequency value of 10 Hz. Finally, Section 7 concludes this work, discussing possible extensions in the direction of completing the simulated data and applying the deep learning model on a larger range of frequencies.

## 2. Related Work

Computational intelligence techniques have been broadly used in NPP operation for many years [3]. For example, artificial neural networks (ANNs) were tested successfully in locating a control rod perturbation from in-core self powered neutron detector (SPND) spectra recorded at Paks-2, a commercial VVER-type pressurized water reactor (PWR) in Hungary [4,5]. In this case, only three detectors were used to detect seven control rod locations. Analysis was performed in the frequency domain with only one perturbation frequency, and a standard three-layered, fully connected feed-forward ANN was used [5] with six nodes in the input layer (three for the auto-spectra and three for the cross-spectra), ten nodes in the hidden layer and seven for the output layer.

Nevertheless, simple architectures, such as the one described above, are not adequate for the more general problem of locating complex perturbations that may cover the whole reactor core, calling for advanced approaches [6]. In recent years, the development of massive parallel processing computation systems at reduced cost (e.g., in the from of graphical or tensor processing units) has permitted the training of much larger ANN architectures on large volumes of data, leading to the introduction of deep learning approaches in NPP operation and safety. In this respect and in the framework of the CORTEX project, a three-dimensional (3D) convolutional neural network (CNN) model in the frequency domain was adapted to the localization problem [7]. Other works analyzed neutron noise signals using recurrent neural networks (RNNs) [8] and long short-term memory (LSTM) units [8,9]. On the other hand, certain time domain analysis approaches firstly compute the wavelet transformation of the neutron noise signals and construct the respective scaleograms. Then, those scaleograms are treated as images by deep CNN architectures that perform the perturbation identification and localization tasks [10,11].

All of the aformentioned methodologies were applied to nuclear reactors in Cartesian geometry for which the transfer function can be calculated by the CORE SIM solver [12,13]. The comparison with real plant data is in progress for some pre-Konvoi nuclear reactors [14]. For the time domain, the simulated perturbation data are generated by the SIMULATE 3K code (S3K) [15]. However, none of these codes can model the hexagonal reactor geometry of the VVER-type reactors. Therefore, the FEMFFUSION code is used instead, after being modified to deal with neutron noise problems in hexagonal geometry, in both the time and frequency domains. In this study, the simulated perturbation data generated by the FEMMFUSION code [16] pertain to a scenario involving a distributed variable-strength neutron absorber over the whole VVER-1000 reactor core. These data are used to train a machine learning model, then this model is used to try to backtrack the source of the perturbation in the core. In a second part, we test the model with real plant measurements from the Temelín NPP in the Czech Republic [17].

## 3. The VVER-1000 Nuclear Reactor at Temelín

In this section, the general characteristics of VVER-1000 reactor are summarized, together with the description of neutron flux measurements.

### 3.1. Reactor Description

**Figure 1:**
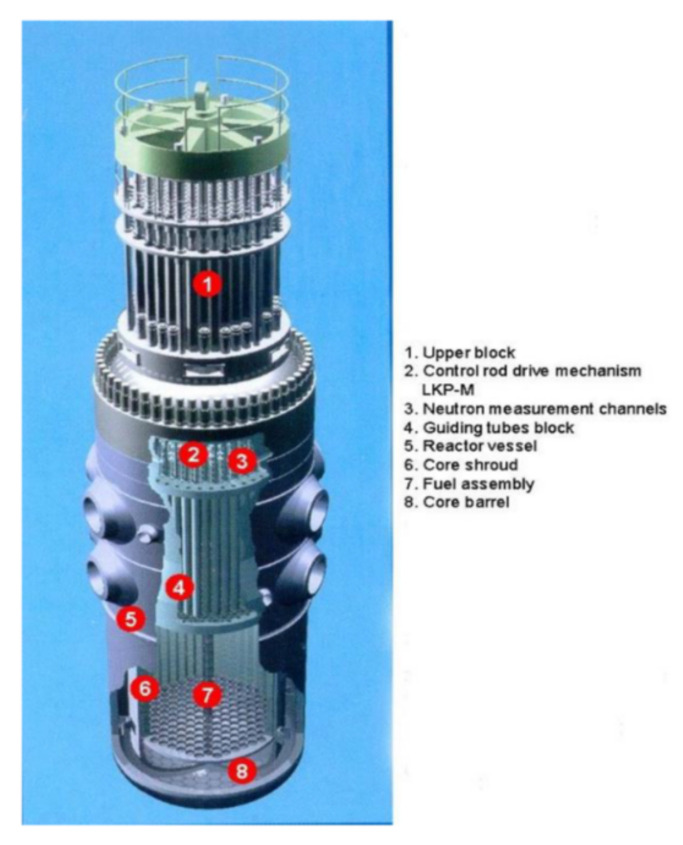
General characteristics of the VVER-1000/320 reactor.

The Temelín NPP is located near Temelín in the Czech Republic [17]. Its technological schema corresponds to a standard Gen III power plant. In the 1990s, alterations to the original design were made by the Westinghouse Electric Corporation, in conjunction with the State Office for Nuclear Safety of the Czech Republic (SÚJB) and the International Atomic Energy Agency (IAEA) in an effort to bring reliability and safety levels into conformance with Western European standards. The entire primary circuit (the nuclear reactor, four loops with steam-generators, circulation pumps, etc.) is in a fully pressurized containment facility, hermetically enclosed in a protection envelope from reinforced concrete. The reactor core contains 163 fuel assemblies, each with 312 fuel rods and 61 regulating rods. The PWR is fueled by uranium dioxide ($UO_{2}$) enriched to an average of 3.5% with the fission isotope ${}^{235}U$. The characteristics of the VVER-1000/320 reactor are presented in Figure 1.

### 3.2. Measurement Methodology

Neutron noise data are measured and gathered with the mobile, in-house distributed measuring test system (DMTS), developed by UJV (nuclear power engineering in Czech Republic), from the standard diagnostic plant reactor vibration monitoring system (RVMS), together with records of technological data. The RVMS diagnostic sensors of each unit include 4 accelerometers on the reactor head flange, 12 ionization chambers placed in three vertical planes and at two horizontal levels, and more than 256 self power neutron detectors (SPNDs) across the whole core in four axial heights in non-uniform radial spreading.

SPND signals are sequentially measured in groups of 16, controlled by means of 19 configuration sets. RVMS measuring chains contain conditioning with isolation and buffer amplifiers, high/low-pass 8-pole Butterworth filters with a minimum of 48 dB per octave roll-off to form anti-aliasing filters in several bandpass ranges. The sampling frequency is up to 1 kHz with 16/12 bits resolution for noise/DC signals. The diagnostic data acquired by DMTS are stored with basic fixed frequency ranges per channel (200 Hz and 300 Hz), with a typical 0.007 Hz lower cut-off frequency, sampled at 1 kHz with 24 bits resolution at the 5 V–10 V output signal of standard NPP diagnostic measuring chains.

**Figure 2:**
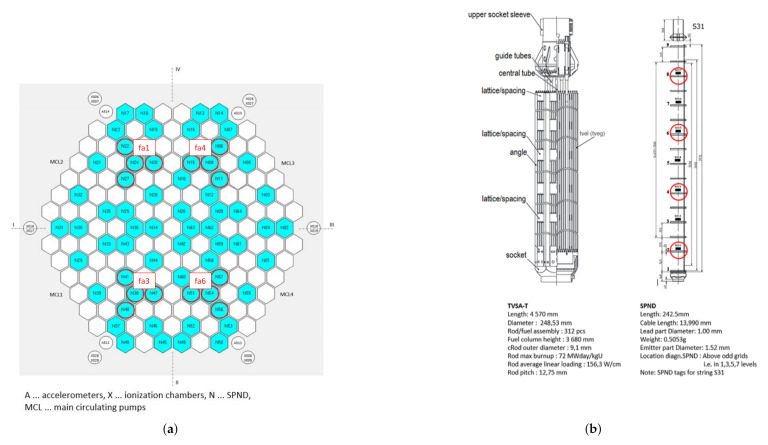
SPND positions in the core of the VVER-1000/320 reactor with data from the *U1C09* cycle. (**a**) Radial detector positions with marked *fa1*, *fa3*, *fa4* and *fa6* sets. (**b**) Vertical detector positions in the TVSA-T fuel assembly.

Figure 2 displays the radial and vertical detector positions with marked fa1, fa3, fa4, and fa6 configuration sets and positions in the TVSA-T fuel assembly.

**Figure 3:**
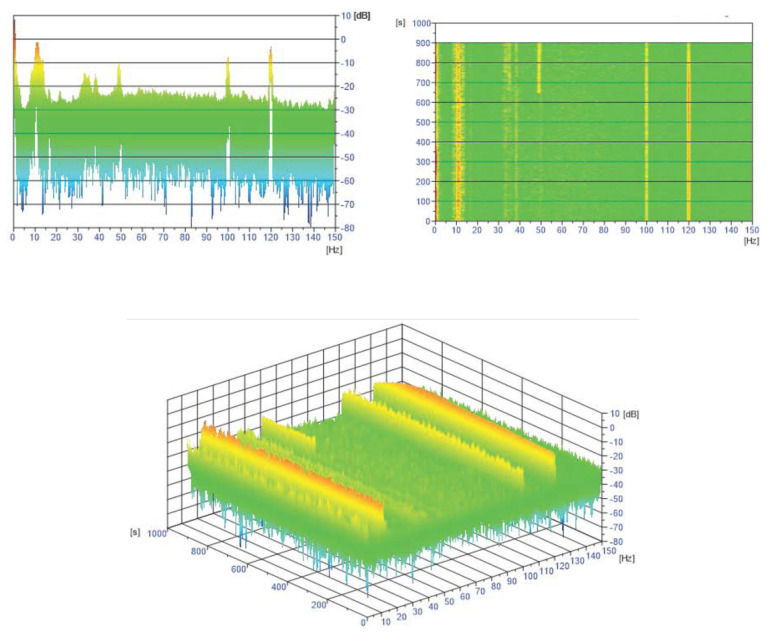
JTFS from the N205 SPND (selected from the *fa1* configuration set) during the U1C09 cycle.

All neutron noise data are shortened to the uniform length of 720,000 samples in order to avoid undesirable transients at the start of the plant measurements. Therefore, there exist uniform 12-min intervals for the processing steps described next. Direct current (DC) components of all neutron noise data in these sets are removed. All ionization chambers and SPND data are normalized to the DC part of the respective signal. Figure 3 exhibits the joint time–frequency spectrograms (JTFSs) of a SPND, selected from the configuration set *fa1* as a typical view in the frequency-time domain.

Data from the beginning of cycle (BOC) *U1C09* were acquired in 19 configuration sets in October 2010, during physical tests of the neutron instrumentation and under strict operational conditions [17,18].

### 3.3. Detectors Used for Structural Health Monitoring

The plant measurements used in this work were acquired in the framework of the study of the control rod insertion reliability. In this respect, the strategy of migrating one assembly in the core through four consecutive fuel cycles (*U1C09*, *U1C10*, *U1C11* and *U1C12*) was followed. At the end of the last cycle, a problem related to incompatible rod insertion (IRI) occurred. Having gathered all appropriate data, it was made possible to investigate and to identify this phenomenon through the neutron noise signals acquired under the same operational conditions in every cycle (i.e., the cycles without trouble) and the cycle where the problem occurred. In order to follow the migration of an assembly in the core, only the neutron noise signals acquired on four detectors groups (*fa1*, *fa2*, *fa3* and *fa4*), as shown in Figure 2a, were taken into account. For this reason, the deep learning architecture (Section 5) is trained four times, learning one model for each detector group (Table 1). Then the idea is to use only the results obtained from one of these groups to determine the location of the problem in the core. In this regard, only the data acquired at the beginning of the first cycle (*U1C09*) are used. The study of the three other cycles is not relevant in the context of this work and, therefore, the IRI problem is not further discussed (hoping, nevertheless, that the methodology initiated in this work serves as intuition for further analyses).

## 4. The Simulated Data

Prior to unfolding, the machine learning algorithms need to be fed with training data, i.e., data where a known perturbation is assumed and the corresponding induced neutron noise at the location of the detectors is estimated. This section deals with the generation of these training sets for hexagonal reactors (in a similar manner, data generation for rectangular geometry reactors is described in [19]). Generation of the training datasets is performed with the FEMFFUSION code in its frequency domain mode [20].

### 4.1. The FEMFFUSION Diffusion Code

**Figure 4:**
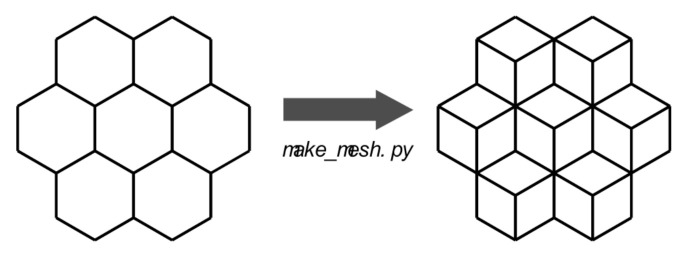
FEMFFUSION code: each hexagon is discretized into 3 quadrilaterals.

FEMFFUSION is an open source finite element code that solves the neutron diffusion approximation [16,21]. This code was extended for the CORTEX project to deal with neutron noise problems in the time domain [22] and in the frequency domain [20], especially for hexagonal reactors. Due to the quantity of the simulations for this work, the calculations are performed in the frequency domain. FEMFFUSION permits any kind of structured and unstructured meshes, as long as they are composed of quadrilaterals (2D) or hexahedrals (3D). In this way, each hexagon of the hexagonal reactor is discretized into 3 quadrilateral cells (Figure 4). A simple extrusion of this geometry is performed so as to account for the height of the reactor.

### 4.2. Neutron Noise Diffusion Equation

FEMFFUSION solves the neutron noise diffusion equation in the multi energy group approximation. The time-dependent neutron diffusion equation can be written as in Equations (1) and (2) below [23]

(1) $$\begin{array}{cc} \left[v^{-1}\right]\frac{\partial \Phi }{\partial t}+L\Phi & =\left(1-\beta \right)M\Phi +\sum_{p=1}^{N_{p}}\lambda_{p}\chi C_{p}, \end{array}$$

(2) $$\begin{array}{cc} \frac{\partial C_{p}}{\partial t} & =\beta_{p}{\left[\nu \Sigma_{f}\right]}^{T}\Phi -\lambda_{p}\;C_{p},\;\;p=1,\;\ldots ,\;N_{p} \end{array}$$

In the two group theory without upscattering, matrices $v^{-1},L,M,\nu \Sigma_{f},\chi$ and Φ are defined as in Equations (3) and (4)

(3) $$\begin{array}{cc} \left[v^{-1}\right] & =\left[\begin{array}{cc} \frac{1}{v_{1}} & 0\\ 0 & \frac{1}{v_{2}} \end{array}\right]\;,\;L=\left[\begin{array}{cc} -\vec{\nabla }\;\cdot \;\left(D_{1}\vec{\nabla }\right)+\Sigma_{a1}+\Sigma_{12} & 0\\ -\Sigma_{12} & \Sigma_{a2} \end{array}\right], \end{array}$$

(4) $$\begin{array}{cc} M & =\left[\begin{array}{cc} \nu \Sigma_{f1} & \nu \Sigma_{f2}\\ 0 & 0 \end{array}\right]\;,\;\nu \Sigma_{f}=\left[\begin{array}{c} \nu \Sigma_{f1}\\ \nu \Sigma_{f2} \end{array}\right]\;,\;\chi =\left[\begin{array}{c} 1\\ 0 \end{array}\right],\;\Phi =\left[\begin{array}{c} \phi_{1}\\ \phi_{2} \end{array}\right]. \end{array}$$

The main unknown of the neutron transport equation is the space- and time-dependent neutron flux in its usual separation in the fast and thermal energy groups $\Phi =[\phi_{1}\left(\vec{r},\;t\right),$$\phi_{2}\left(\vec{r},\;t\right){]}^{T},$ and the neutron precursor concentration $C_{p}\left(\vec{r},\;t\right)$ for each neutron precursor group *p*. The total delayed neutrons fraction is $\beta =\sum_{p=1}^{N_{p}}\beta_{p}$. $\Sigma_{a1}$ and $\Sigma_{a2}$ are the fast and thermal absorption cross sections that quantify the probability of a fast or a thermal neutron to be absorbed by a nucleus per centimeter of neutron travel, respectively. In the same way, $\Sigma_{f1}$ and $\Sigma_{f2}$ are the probabilities of a fission reaction produced by a fast or a thermal neutron per centimeter of neutron travel. ν is the average number of neutrons released per fission, and $\Sigma_{12}$ is the probability of a scattering interaction per centimeter of fast neutron travel. These cross sections are determined by the materials of the reactor. All other quantities have their usual meaning in the nuclear engineering area [23]. The first order neutron noise theory [24] splits all time-dependent terms of Equations (1) and (2), $X(\vec{r},t)$, into their mean (or static) values $X_{0}\left(\vec{r}\right)$ and their fluctuation around the mean $\delta X(\vec{r},t)$ (Equation (5))

(5) $$X\left(\vec{r},t\right)=X_{0}\left(\vec{r}\right)+\delta X\left(\vec{r},t\right)$$

This separation stands for (i) The neutron operators, L and M; (ii) The materials cross sections $\Sigma_{a}$, $\Sigma_{f}$, $\Sigma_{12}$; (iii) The concentration of delayed neutron precursors; (iv) $C_{p}$; (v) The neutron flux, Φ.

Then, the following assumptions are made:

1. Fluctuations are assumed to be small.
  $$|\delta X\left(\vec{r},t\right)|\ll X_{0}\left(\vec{r}\right),\;\forall \left(\vec{r},t\right).$$
2. The transient is assumed to be stationary.
  $$\langle \delta X(\vec{r},t)\rangle =0,\;\forall \left(\vec{r},t\right).$$
3. Second-order terms are neglected.
  $$\delta X\left(\vec{r},t\right)\;\cdot \;\delta Y\left(\vec{r},t\right)\approx 0.$$

Applying the neutron noise separation from Equation (5) into Equations (1) and (2), removing the second-order terms and performing a Fourier transformation, the frequency-domain neutron noise equation becomes (Equation (6))

(6) $$A\delta \Phi =B\Phi_{0}.$$

In the usual two group approximation,

$$A=\left[\begin{array}{cc} \frac{i\omega }{\Sigma_{a1}+\Sigma_{12}}-\gamma \nu \Sigma_{f1}\; & -\gamma \nu \Sigma_{f2}\\ -\Sigma_{12}\; & \frac{i\omega }{\Sigma_{a2}} \end{array}\right],\;B=\left[\begin{array}{cc} -\delta \Sigma_{a1}-\Sigma_{12}+\gamma \delta \Sigma_{f1}\; & +\gamma \delta \Sigma_{f2}\\ \delta \Sigma_{12}\; & -\delta \Sigma_{a2} \end{array}\right],$$

where

$$\gamma =\left(1-\beta \right)+\sum_{p=1}^{N_{p}}\frac{\lambda_{p}\beta_{p}}{i\omega +\lambda_{p}}.$$

It can be seen that the neutron noise Equation (6) is an inhomogeneous equation with complex-value quantities. The application of the continuous Galerkin finite element discretization [25] leads to an algebraic system of equations with complex values and the block structure of Equation (7) below

(7) $$\left(\begin{array}{cc} {\tilde{A}}_{11}\; & {\tilde{A}}_{12}\\ {\tilde{A}}_{21}\; & {\tilde{A}}_{22} \end{array}\right)\left(\begin{array}{c} \delta {\tilde{\phi }}_{1}\\ \delta {\tilde{\phi }}_{2} \end{array}\right)=\left(\begin{array}{cc} {\tilde{B}}_{11} & {\tilde{B}}_{12}\\ {\tilde{B}}_{21} & {\tilde{B}}_{22} \end{array}\right)\left(\begin{array}{c} {\tilde{\phi }}_{0,1}\\ {\tilde{\phi }}_{0,2} \end{array}\right),$$

where $\delta {\tilde{\phi }}_{1}$ and $\delta {\tilde{\phi }}_{2}$ are the algebraic vectors of weights associated with the fast and thermal neutron noise in the frequency domain. This complex value system has to be solved after the steady-state problem that calculates the algebraic vectors of weights, associated with the fast and thermal static neutron flux, ${\tilde{\phi }}_{0,1}$ and ${\tilde{\phi }}_{0,2}$, respectively. The related static eigenvalue problem must be solved with the same spatial discretization as the frequency domain neutron noise to obtain coherent results. This system is transformed into an equivalent system of equations with real values, and the sparse system of Equation (7) is solved using a biconjugate gradient stabilized method [26], with an incomplete LU decomposition [27] employed as preconditioner.

**Figure 5:**
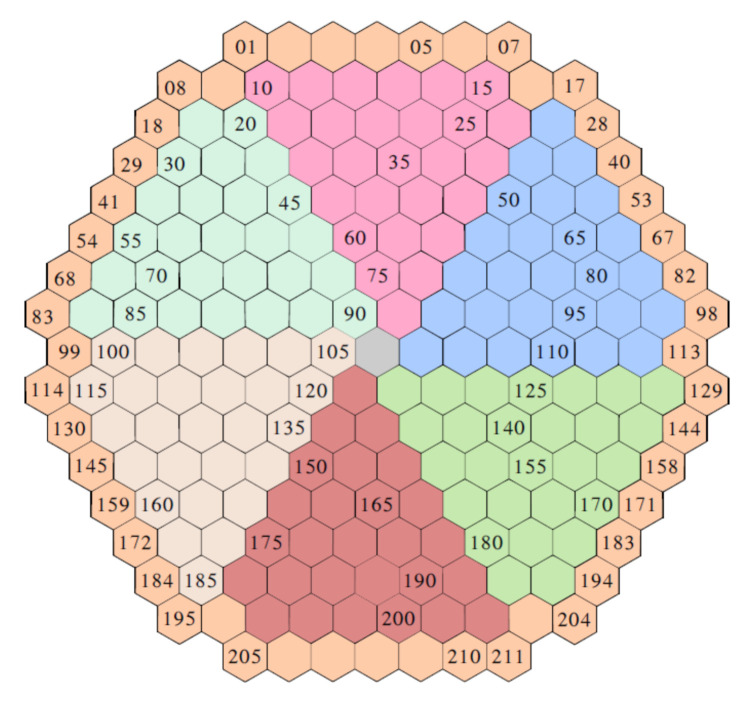
Numbering of hexagonal cells in the VVER-1000 reactor.

Each simulation calculates the thermal relative neutron noise $\delta \phi_{2}/\phi_{0,2}$ at each detector position. The VVER-1000 reactor is modeled using 221 vertical assemblies discretized in 50 planes, summing to a total of 10,550 hexagonal cells. The hexagons are sorted using a left to right and up to down numbering (Figure 5). Successive planes use a correlative numbering.

### 4.3. Generic Absorber of Variable Strength

The scenario being considered is a Dirac-like perturbation at the hexagonal cell, directly expressed as a perturbation of the macroscopic absorption cross-sections. This scenario is particularly important since it can be used to localize a generic type of perturbation that does not fit any special category. The perturbation inserted at each simulation is set to $\delta \Sigma_{a1}\left(c,\omega \right)=0.1$ and $\delta \Sigma_{a2}\left(c,\omega \right)=0.1$, where *c* is the hexagonal cell and ω is the angular frequency of the perturbation. Three perturbation frequencies are considered: (i) 0.1 Hz; (ii) 1 Hz; (iii) 10 Hz.

In this way, 31,650 simulations for the VVER-1000 reactor are performed. Due to the volume of the simulations, all calculations are made with linear finite elements, where each simulation takes approximately 30 s, using one processor.

## 5. The Machine Learning Model

Based on the available data (simulated and real), the objective of the machine learning model is to perform an *identification* task: that is, to identify if any perturbations occur with respect to each fuel assembly. For this purpose, the chosen model is firstly trained on all of the available simulated data, where it is known in advance at which fuel assembly the perturbation occurs, thereby forming a supervised classification problem. Once the training phase completes, model performance is validated on plant measurements in an effort to identify possible perturbation locations.

### 5.1. Model Architecture

**Figure 6:**
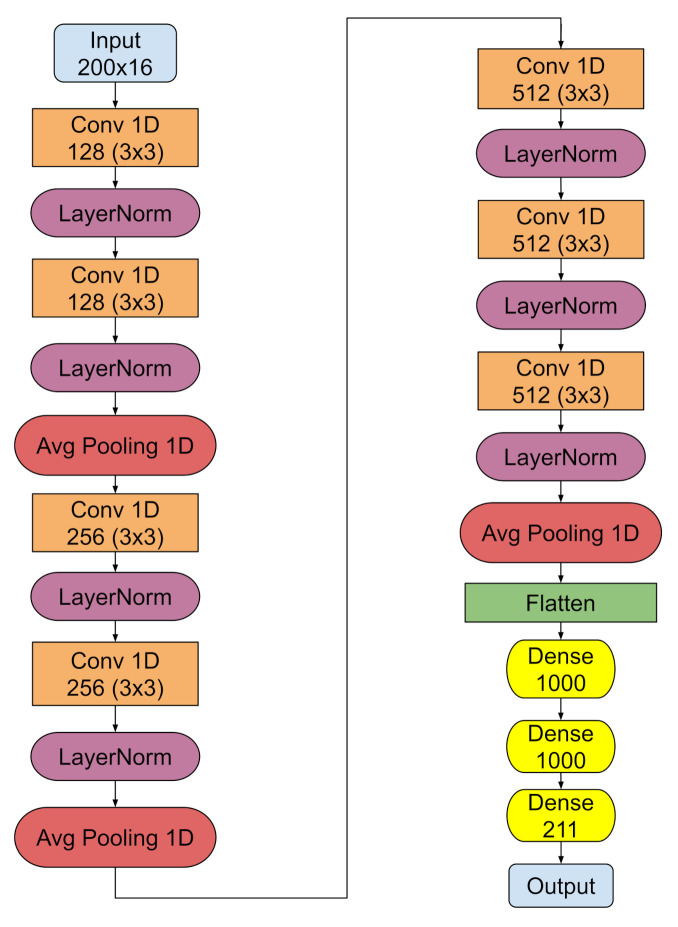
The machine learning model.

Figure 6 outlines the optimal model architecture. It is a deep convolutional neural network, whose input is comprised of 16 in-core signals. More precisely and in order to align the simulated data with the plant measurements, four groups of 16 signals are created, as illustrated on Table 1 below, thereby training four distinct models.

Each detector signal has a length of 200 timesteps. The simulated data files contain the result of the calculated complex value, which gives the characteristics of the vibration arriving on the detector for each radial and axial location. The transformation from frequency to the time domain is realized according to Equation (8) below

(8) $$\frac{\delta \phi (x,t)}{\phi_{0}\left(x\right)}=\frac{\left|\delta \phi \left(x,\omega_{p}\right)\right|}{\phi_{0}\left(x\right)}\sin\left(\omega_{p}t+\arg\left(\delta \phi (x,\omega_{p})\right)\right),$$

where $\omega_{p}$ is the angular frequency of the perturbation and thus the dominant frequency of the neutron noise flux.

Additionally, the simulated data are normalized (divided by the global standard deviation). Concerning the plant measurements, they are already on the time domain. The size of the signals are decimated by a factor of 10, in order to speed up calculations for model training, while at the same time retaining the necessary frequency content for the physical analysis. Then, different sample sizes of 200 timesteps are chosen during training. Finally, two approaches are followed prior to providing the plant measurements to the machine learning model. The first is to provide the raw signal (after detrending and normalization), while the second is to perform an additional preprocessing step: the application of a band-pass filter on specific frequencies, only those that were simulated and used for model training.

As pictured in Figure 6, the input signals undergo a number of consecutive feature extraction steps that are comprised of one-dimensional convolutions, followed by normalization layers [28] that normalize the input to the next layer by computing its mean and variance. Then, every two convolutions, the signal is averaged by an one-dimensional average pooling layer. The first two convolutions involve 128 feature maps that are produced by kernels of size 3×3, followed by two convolutions of 256 feature maps of equal sized kernels and then of two final convolutions of 512 feature maps. Then, the output of the final feature extraction step is flattened (green rectangle) and provided to the fully connected component at the end of the architecture (three yellow rectangles) that performs the localization task. This component consists of three dense layers, with the first two having 1000 neurons each, while the last one has 211, corresponding to each fuel assembly within the core. The overall architecture is trained on the labeled simulated data, using the Adam optimization technique [29] for 200 epochs for the four different groups discussed above.

According to the available simulated data, it would be possible to locate every perturbation at 211×50 locations because for each radial position, 50 axial levels in the core are calculated. However, this would produce a 10,550 output vector (one-hot encoded for every axial and radial combination). At this point of our research, we are primarily interested in identifying the perturbations at the fuel assembly level (Figure 5); therefore, the machine learning model returns the radial position at each given frequency, regardless of the axial position where the perturbation actually occurs. This observation is necessary in order to understand the results obtained by the machine learning models in Section 6.2.

## 6. Results and Discussion

Currently, the simulated data are created for three frequencies and only for the irradiation conditions related to the first cycle *U1C09* (Section 3.2 and Section 4.3). Validation scores regarding the capability of the trained models to localize the anomalies are being reported and subsequently the models are being tested on actual plant measurements. In the discussion section (Section 6.2), it is demonstrated that the proposed methodology exhibits better results at 10 Hz, while performance at the very low frequencies (0.1 Hz and 1 Hz) is not as optimal.

### 6.1. Results

#### 6.1.1. The Efficiency of the Trained Models

Table 2 summarizes the training accuracy of the four different models trained on the respective detector subsets of Table 1 (Section 5) on the simulated data. The achieved performance means that the machine learning models are capable of identifying the fuel assembly where the perturbation occurs more than 9 in 10 times.

#### 6.1.2. Prediction on the Plant Measurements

**Figure 7:**
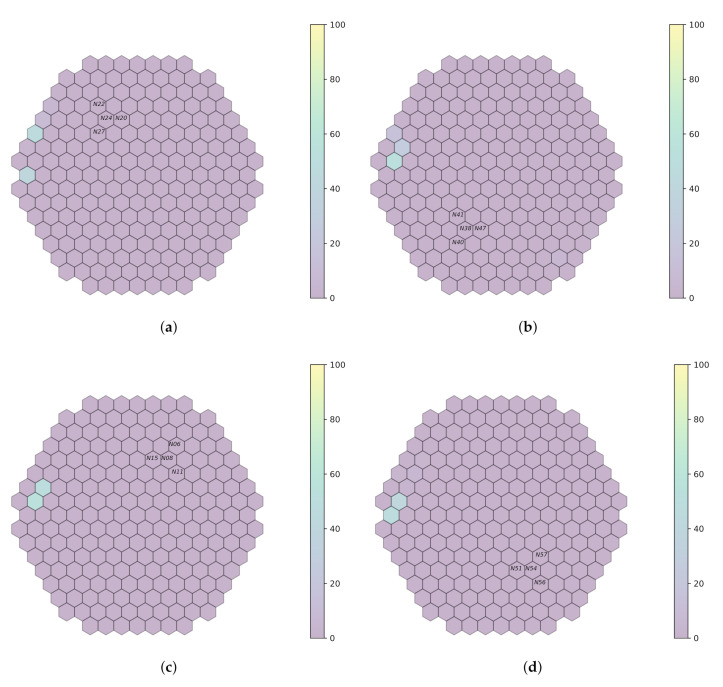
Prediction on plant measurements when no filter is applied (sampling rate 100 Hz). (**a**) *fa1*. (**b**) *fa3*. (**c**) *fa4*. (**d**) *fa6*.

**Figure 8:**
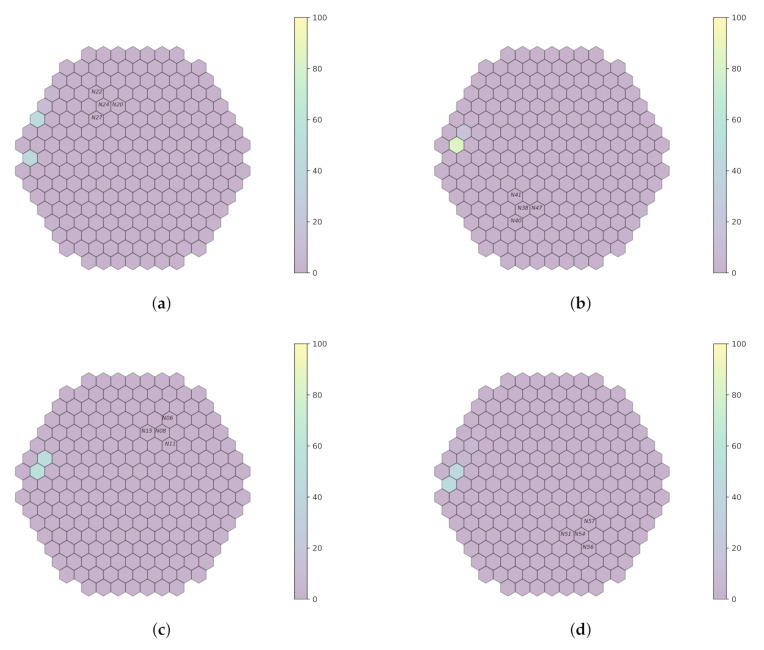
Prediction on plant measurements for filtered bandwidth [0;1.1]Hz and sampling rate of 100 Hz (Section 6.2.2). (**a**) *fa1*. (**b**) *fa3*. (**c**) *fa4*. (**d**) *fa6*.

**Figure 9:**
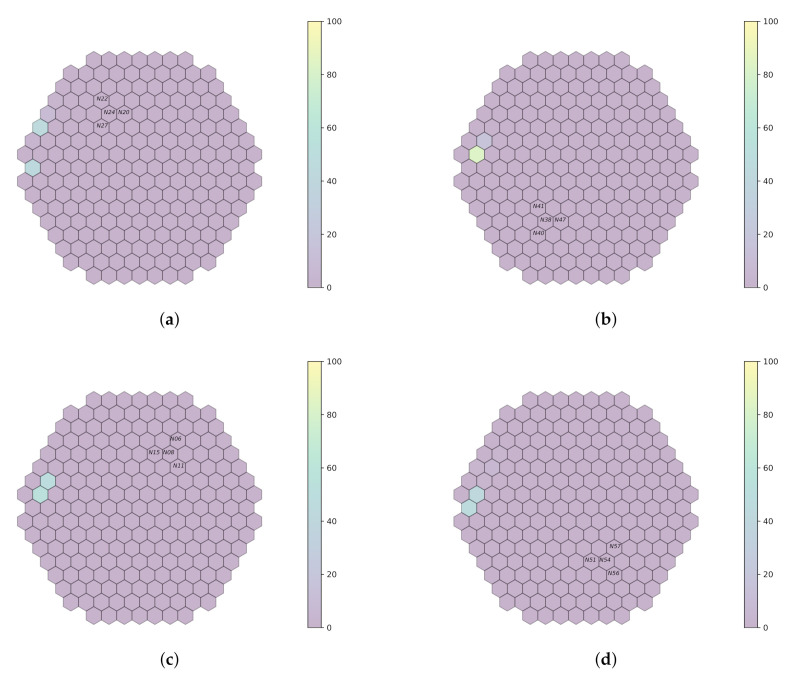
Prediction on plant measurements for filtered bandwidth [0;2]Hz and sampling rate of 100 Hz. (**a**) *fa1*. (**b**) *fa3*. (**c**) *fa4*. (**d**) *fa6*.

Every trained model in the simulated data is validated on plant measurements from the Temelín reactor, at 0.1 Hz, 1 Hz and 10 Hz, for a limited number of detectors. Initially, the models are tested on raw measurements (that is, without filtering). Then, a band-pass filter is applied around each simulated frequency used during model training. Figure 7, Figure 8 and Figure 9 outline system performance on the actual plant measurements, thanks to a visual representation, which gives an assessment of the localization probabilities for the perturbation.

### 6.2. Discussion

#### 6.2.1. The Other Available Measurements in the Core

**Figure 10:**
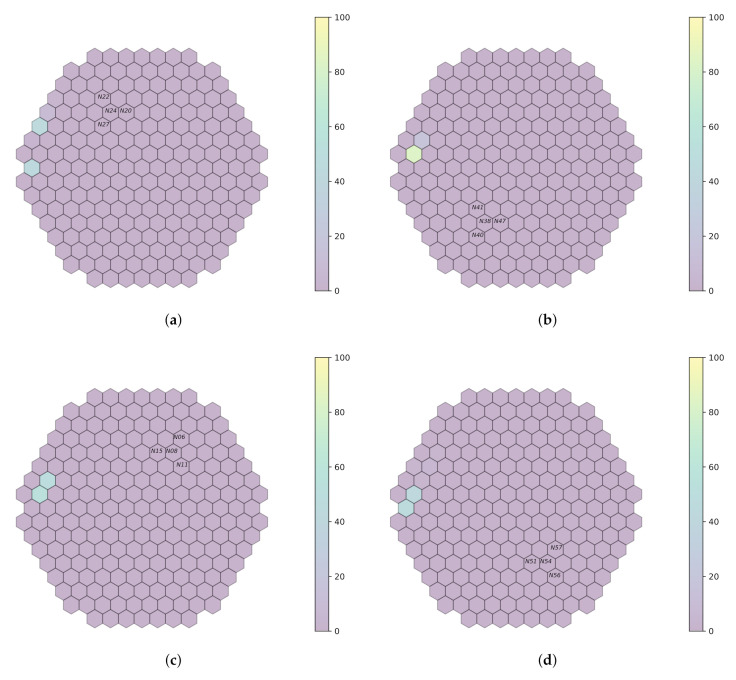
Prediction on plant measurements for filtered bandwidth [9;11]Hz and sampling rate of 100 Hz. (**a**) *fa1*. (**b**) *fa3*. (**c**) *fa4*. (**d**) *fa6*.

**Figure 11:**
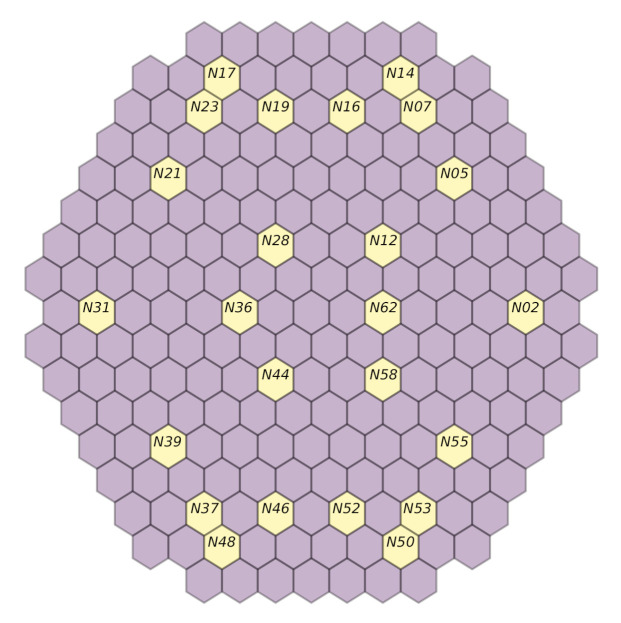
Available measurements for the *U1C09* cycle, used to verify the machine learning performance.

As indicated in Section 3.3, data acquired at the beginning of the cycle *U1C09* are used, while the focus is around only three frequencies for which simulated data are available (Section 4.3). In order to be able to reason on the results shown in Figure 7, Figure 8, Figure 9 and Figure 10, the presence or absence of the perturbation at the predicted frequency and location needs to be verified. This is achieved by examining the available measurements acquired by the neutron detectors in the core and, more specifically, for the closest assembly to the radial location predicted by the models. Afterwards, the spectrum analysis of the signals acquired by the detectors on all the axial locations for this given radial location will provide an indication of validating the prediction; it needs to be verified whether a peak effectively exists at the perturbation frequency used by the model to realize the prediction on all axial locations. Regarding the first cycle (*U1C09*), the available radial locations are displayed in Figure 11. Additionally, for every model, the signals acquired on the detectors not considered by the models may be used, e.g., if the performance of the *a1* model is evaluated, then the detector signals of groups *fa3*, *fa4* and *fa6* may be employed.

#### 6.2.2. Some Considerations on the Frequency Resolution

It is known that the signal length limits the frequency resolution which can be expressed as $\Delta f=\frac{Fs}{n}$, where Fs is the sampling frequency and *n* the size of the signal. Plant measurements are acquired for a duration of 12 min, with a sampling frequency of 1 kHz (Section 3.2). Therefore, the frequency resolution is expected to be equal to $14\times 10^{-4}$ Hz. In practice, the detector signals are decimated by a factor of ten in order to speed up the calculation time, resulting in 72,000 samples per signal for model training. This choice is feasible since the range of frequencies of interest for neutron noise analysis does not exceed 50 Hz. In an effort to further reduce training time, only 200 samples from each signal are actually considered (Section 5.1). This proves to be a good tradeoff, but in this case, the frequency resolution is limited to 0.5 Hz, excluding perturbations around 0.1 Hz. For the 1 Hz and 10 Hz cases, 200 points are sufficient to describe 2 and 20 periods of the sinusoid, respectively, which is good for the latter frequency and acceptable for the former.

#### 6.2.3. Spectra Observation of the Other Available Measurements to Validate the Prediction

**Figure 12:**
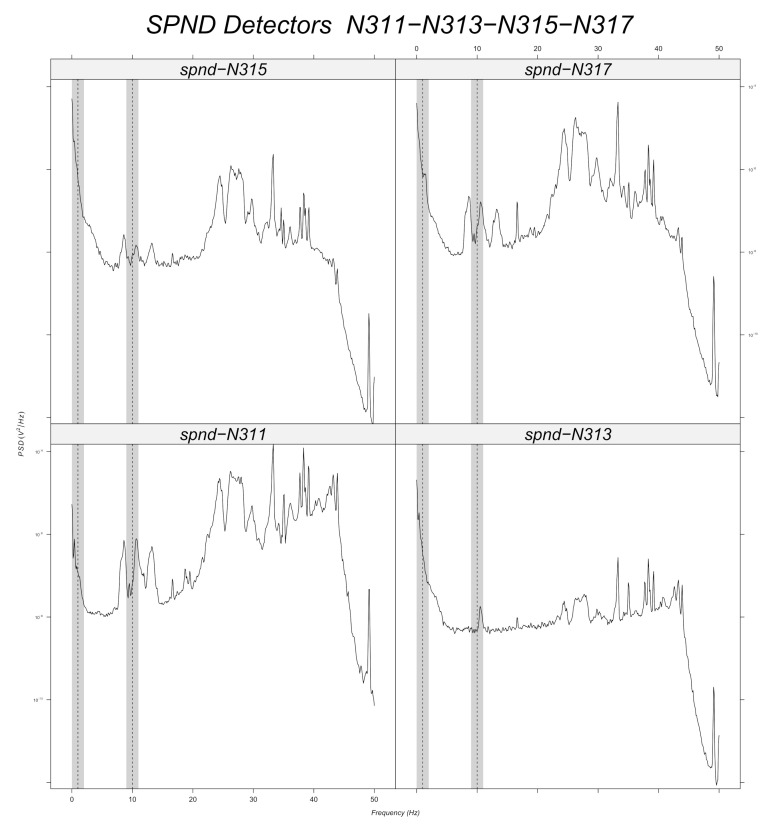
Power spectral density measured at radial location N31 (for all axial positions).

**Figure 13:**
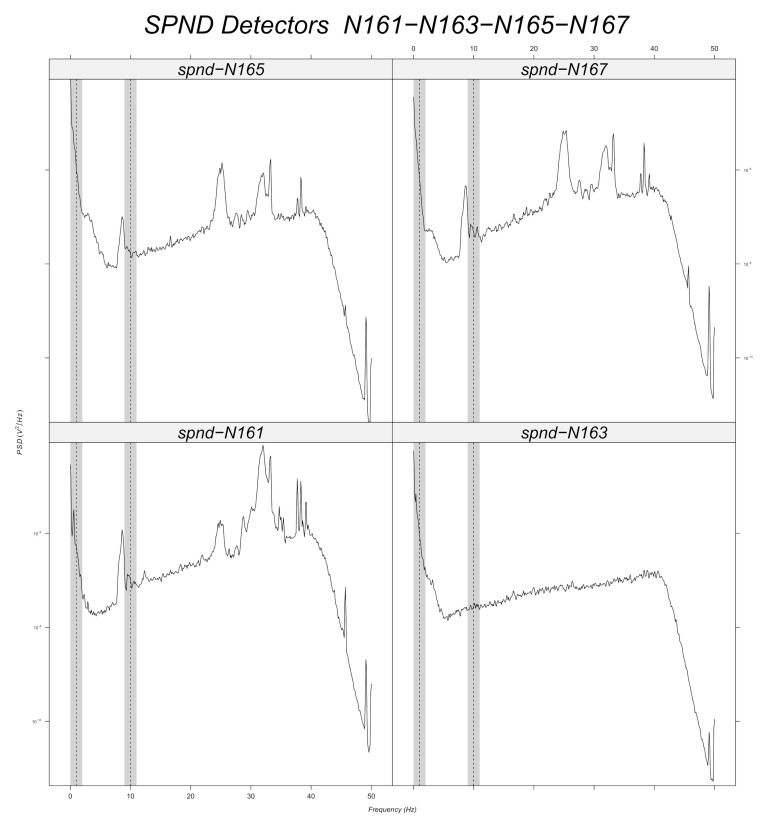
Power spectral density measured at radial location N16 (for all axial positions).

**Figure 14:**
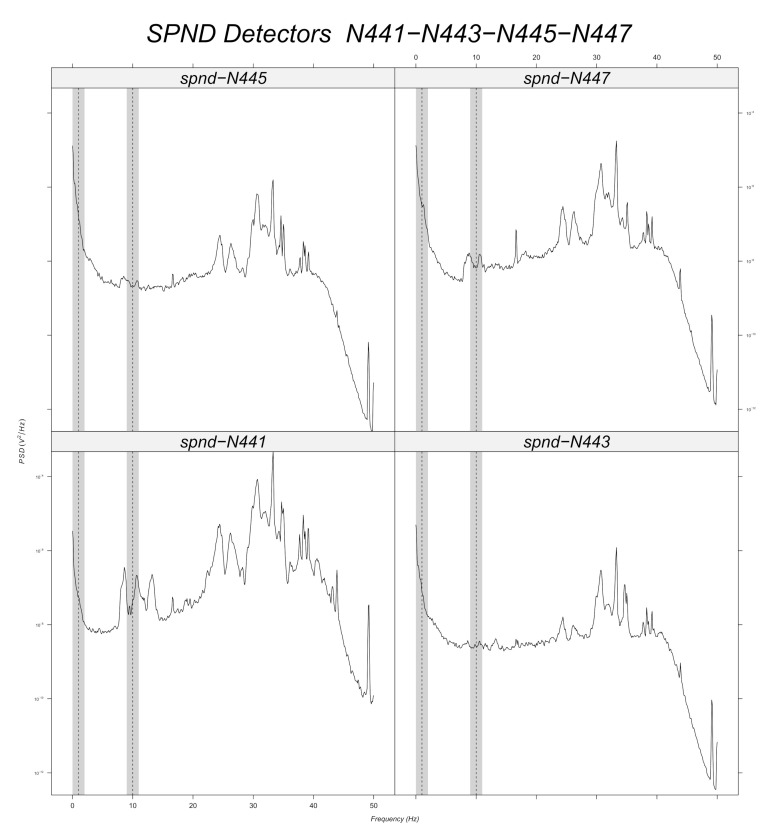
Power spectral density measured at radial location N44 (for all axial positions).

**Figure 15:**
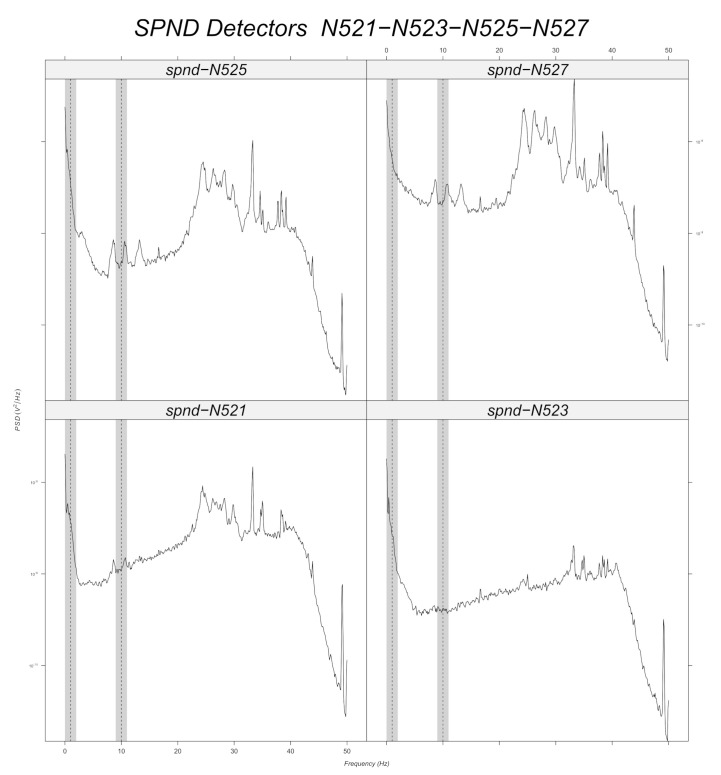
Power spectral density measured at radial location N52 (for all axial positions).

**Figure 16:**
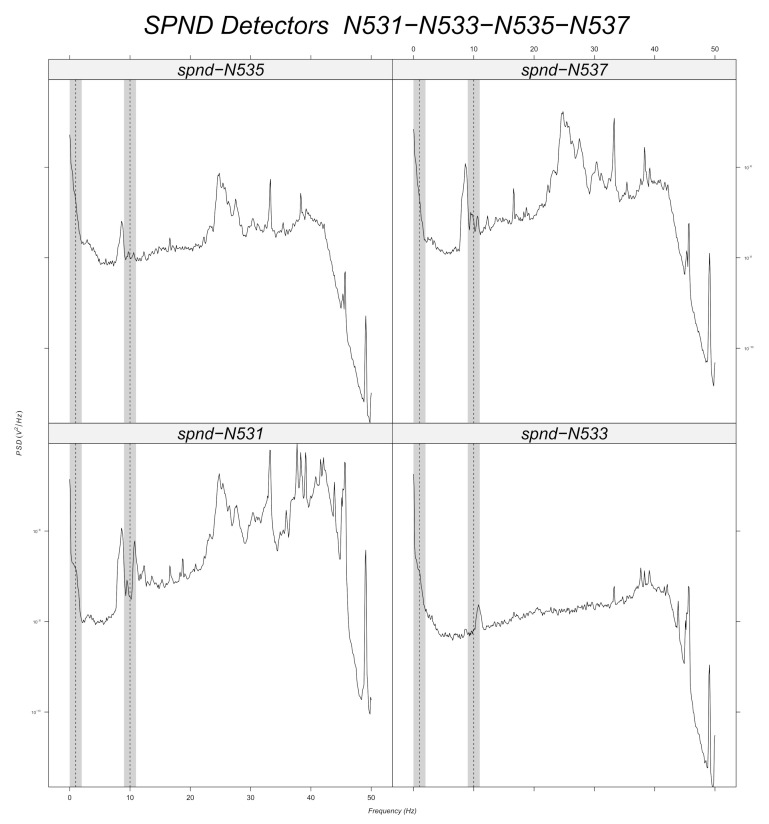
Power spectral density measured at radial location N53 (for all axial positions).

**Figure 17:**
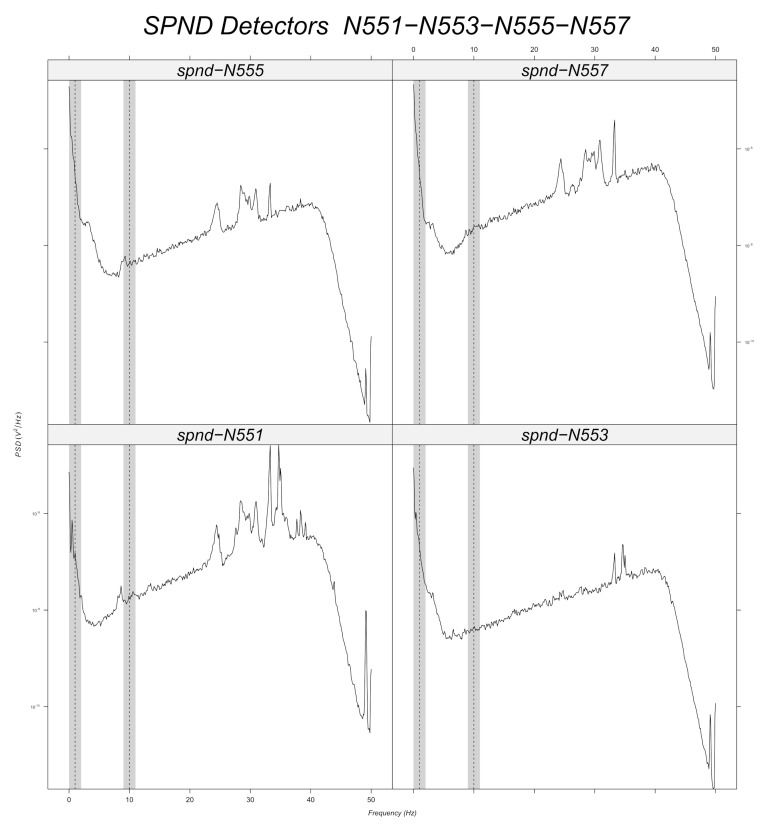
Power spectral density measured at radial location N55 (for all axial positions).

Prior to machine learning model training, a band-pass filter is applied to the simulated data around every frequency with a tolerance of 1 Hz (Section 6.2.2). Signal stationarity is also important, as the initial signals are reduced to 200 samples. This choice is verified for 10 Hz, as it can be seen in the example of Figure 3 for the N205 detector. Figure 12, Figure 13, Figure 14, Figure 15, Figure 16 and Figure 17 displays the spectra calculated on the signal evolution of the available detectors not used in model training.

In Figure 12, the radial position of detector N31 is marked, as the frequency value of 10 Hz is always present in the spectrum along all axial positions (N311, N313, N315, N317). Additionally, 1 Hz is highlighted around every frequency of the band-pass filters in order to determine its presence or absence. Radial position N31 is also close to a predicted perturbation location by the machine learning model (Figure 10). Therefore, it could be said that the performance of machine learning models is satisfactory for the 10 Hz case.

For the radial position N62, a frequency value of 10 Hz is observed at the first axial position (N621, Figure 10), but not for the others. That is also the case for the radial locations N44, N52 and N53 (cf. Figure 14, Figure 15 and Figure 16). For the other shapes, there is no ambiguity, for instance with the radial locations N55 (cf. Figure 17, bottom right side of the core) where there is absolutely no peak around 10 Hz or the radial location N16 (cf. Figure 13, upper right side) where the peaks around 10 Hz are very weak at two axial levels. The same kind of observation is also valid for the groups of detector signals not used during model training.

For the 1 Hz case, interpretation is not so obvious since the shape of the spectra in the vicinity of 1 Hz are all identical and the machine learning models predict only one location on the left. At 2 Hz, the noise rapidly increases due to a so-called $\frac{1}{f}$ noise, which is characteristic of neutron noise measurements in power reactors because of the population of the delayed neutrons emitted after nuclear fission by the fission products. The shape of the power spectral density at low frequencies is the same for all detectors, except for N317, where there is a peak near 1 Hz, which can explain the prediction of the model in the vicinity of the N31 detector radial location (Figure 9). However, previously, for the same kind of observation with the peak at 10 Hz in the spectrum of the N621 detector, the machine learning models did not exhibit the same classification results. This behavior needs to be further studied but it could be attributed to the fact that the frequency of 1 Hz is not very well represented by only 200 points over time (only two periods of the sinusoid, as discussed in Section 6.2.2).

Finally, when no filter is applied to the simulated data (Figure 7), predictions are coherent in the sense that they are similar to predictions obtained in the case of the band-pass filter around the frequency value of 10 Hz. The reliability of the obtained results for the three band-pass filters is summarized on Table 3.

## 7. Conclusions

In this work, machine learning models were employed in order to identify and localize perturbations in plant measurements originating from the Temelín VVER-1000 NPP in the Czech Republic. The FEMFFUSION code allowed the generation of simulated data in hexagonal geometry, associated with a Dirac-type perturbation in the core. A one-dimensional deep neural network was trained on these data in order to predict the radial position of the perturbations. It was demonstrated that is possible to reach some reliable conclusions on the radial localization, when the perturbation is around 10 Hz. Because of certain performance considerations during machine learning training, the frequency resolution of the spectral analysis had to be limited, which resulted in non-optimal performance for the 0.1 Hz and 1 Hz frequency values.

This study can be extended in a number of ways. Firstly, new simulated data can be generated for frequencies greater than 5 Hz, that is to say, over the very low noise frequencies in $\frac{1}{f}$. Then, machine learning models will be trained for every group of detectors, for the irradiation conditions specific to every fuel cycle until the incompatible rod insertion (IRI) phenomenon. If the said phenomenon can be related to a frequency in this frequency range, it will be possible to propose an online methodology that detects the radial location of its occurrence.

Additionally, the part of the spectrum at very low frequencies (less than 2 Hz) needs to be better analyzed. This may be achieved by increasing the sample size considered for model training (currently at 200 samples). This would result in an increase of the frequency resolution and as a consequence, it would improve the spectrum description at very low frequencies. Finally, a possible extension of this work is to consider the axial location of the occurring perturbations, apart from the radial one.

## Figures and Tables

**Table 1:** Detector signal groups (letter *N* designating internal detectors, the first two digits their radial position and the last digit the axial position).

Group Name	Detectors
*fa1*	N221, N223, N225, N227, N241, N243, N245, N247, N271, N273,
	N275, N277, N201, N203, N205, N207
*fa3*	N411, N413, N415, N417, N381, N383, N385, N387, N471, N473,
	N475, N477, N401, N403, N405, N407
*fa4*	N151, N153, N155, N157, N081, N083, N085, N087, N061, N063,
	N065, N067, N111, N113, N115, N117
*fa6*	N571, N573, N575, N577, N541, N543, N545, N547, N511, N513,
	N515, N517, N561, N563, N565, N567

**Table 2:** Training accuracy on the different detector signal groups.

Group Name	*fa1*	*fa3*	*fa4*	*fa6*
Accuracy	93.25%	93.95%	93.74%	93.26%

**Table 3:** Reliability of the obtained results for the three band-pass filters.

Model Characteristic	0.1 Hz	1 Hz	10 Hz
Prediction accuracy of the radial location	low	medium	high
Spectral resolution	not well described	partially described	well described

## Data Availability

The data presented in this study are available on request from the corresponding author. The data are not publicly available due to non-disclosure agreements signed in the framework of the CORTEX project.

## Abbreviations

The following abbreviations are used in this manuscript:

ANN	Artificial Neural Network
APSD	Auto-Power Spectral Density
BOC	Beginning of Cycle
CNN	Convolutional Neural Network
CPSD	Cross-Power Spectral Density
DC	Direct Current component
DMTS	Distributed Measuring Test System
FA	Fuel Assembly
IAEA	International Atomic Energy Agency
IRI	Incompatible Rod Insertion
JTFS	Joint Time–Frequency Spectrogram
LSTM	Long Short-Term Memory unit
ML	Machine Learning
NPP	Nuclear Power Plant
PWR	Pressurized Water Reactor
RNN	Recurrent Neural Network
RVMS	Reactor Vibration Monitoring System
SPND	Self Power Neutron Detector
SÚJB	State Office for Nuclear Safety (Czech Republic)
UJV	Nuclear power engineering in Czech Republic (ÚJV Řež)

## References

[1] Ribeiro F.D.S., Calivá F., Chionis D., Dokhane A., Mylonakis A., Demazière C., Leontidis G., Kollias S. Towards a Deep Unified Framework for Nuclear Reactor Perturbation Analysis. Proceedings of the 2018 IEEE Symposium Series on Computational Intelligence (SSCI).

[2] (2017). Noise-Based Core Monitoring and Diagnostics—Overview of the Cortex Project.

[3] Tambouratzis T., Giannatsis J., Kyriazis A., Siotropos P. (2020). Applying the Computational Intelligence Paradigm to Nuclear Power Plant Operation: A Review (1990–2015). Int. J. Energy Optim. Eng..

[4] Pázsit I., Glöckler O. (1988). On the Neutron Noise Diagnostics of Pressurized Water Reactor Control Rod Vibrations. III. Application at a Power Plant. Nucl. Sci. Eng..

[5] Pázsit I., Garis N.S., Glöckler O. (1996). On the Neutron Noise Diagnostics of Pressurized Water Reactor Control Rod Vibrations—IV: Application of Neural Networks. Nucl. Sci. Eng..

[6] Karlsson J.H., Pázsit I. (1999). Localisation of a channel instability in the Forsmark-1 boiling water reactor. Ann. Nucl. Energy.

[7] Demazière C., Mylonakis A., Vinai P., Durrant A., De Sousa Ribeiro F., Wingate J., Leontidis G., Kollias S. (2021). Neutron noise-based anomaly classification and localization using machine learning. EPJ Web Conf..

[8] Durrant A., Leontidis G., Kollias S. (2019). 3D convolutional and recurrent neural networks for reactor perturbation unfolding and anomaly detection. EPJ Nucl. Sci. Technol..

[9] Ioannou G., Tagaris T., Alexandridis G., Stafylopatis A. (2021). Intelligent techniques for anomaly detection IN nuclear reactors. EPJ Web Conf..

[10] Tagaris T., Ioannou G., Sdraka M., Alexandridis G., Stafylopatis A. (2019). Putting Together Wavelet-Based Scaleograms and Convolutional Neural Networks for Anomaly Detection in Nuclear Reactors. Proceedings of the 2019 3rd International Conference on Advances in Artificial Intelligence (ICAAI 2019).

[11] Tasakos T., Ioannou G., Verma V., Alexandridis G., Dokhane A., Stafylopatis A. Deep Learning-Based Anomaly Detection in Nuclear Reactor Cores. Proceedings of the International Conference on Mathematics & Computational Methods Applied to Nuclear Science & Engineering (M&C 2021).

[12] Demazière C. (2011). CORE SIM: A multi-purpose neutronic tool for research and education. Ann. Nucl. Energy.

[13] Mylonakis A., Vinai P., Demazière C. (2021). CORE SIM+: A flexible diffusion-based solver for neutron noise simulations. Ann. Nucl. Energy.

[14] Ioannou G., Tasakos T., Mylonakis A., Alexandridis G., Demaziere C., Vinai P., Stafylopatis A. Feature Extraction and Identification Techniques for the Alignment of Perturbation Simulations with Power Plant Measurements. Proceedings of the International Conference on Mathematics & Computational Methods Applied to Nuclear Science & Engineering (M&C 2021).

[15] Studsvik SIMULATE-3K. https://www.studsvik.com/what-we-do/products/simulate3-k/.

[16] Vidal-Ferràndiz A., Carreño A., Ginestar D., Verdú G. (2020). FEMFFUSION: A Finite Element Code for Nuclear Reactor Modelling. https://www.femffusion.imm.upv.es/.

[17] Stulik P., Bem M., Tschiesche J., Machek J. (2020). CORTEX WP4 Progress Report on subtask T4.2.3. Progress Report ver.01, Core Monitoring Techniques and Experimental Validation and Demonstration (CORTEX), Horizon 2020 EU Framework Programm (No. 754316).

[18] Stulik P., Torres L., Montalvo C., García-Berrocal A., Salazar C., Alexandridis G., Tabouratzis T., Machek J., Pantera L., Bem M. (2019). CORTEX Deliverable 3.3: Development of advanced signal processing techniques and evaluation results. Deliverable D3.3, Core Monitoring Techniques and Experimental Validation and Demonstration (CORTEX), Horizon 2020 EU Framework Programm (No. 754316).

[19] Dokhane A., Mylonakis A. (2019). CORTEX Deliverable 3.2: Description of simulated data. Deliverable D3.2, Core Monitoring Techniques and Experimental Validation and Demonstration (CORTEX), Horizon 2020 EU Framework Programm (No. 754316).

[20] Vidal-Ferràndiz A., Ginestar D., Carreño A., Verdú G., Demazière C. (2021). A finite element method for neutron noise analysis in hexagonal reactors. EPJ Web Conf..

[21] Vidal-Ferrandiz A., Fayez R., Ginestar D., Verdú G. (2014). Solution of the Lambda modes problem of a nuclear power reactor using an h–p finite element method. Ann. Nucl. Energy.

[22] Vidal-Ferràndiz A., Carreño A., Ginestar D., Verdú G. (2020). A block Arnoldi method for the SPN equations. Int. J. Comput. Math..

[23] Stacey P.W.M. (2007). Nuclear Reactor Physics.

[24] Pázsit I., Demazière C., Cacuci D.G. (2010). Noise Techniques in Nuclear Systems. Handbook of Nuclear Engineering.

[25] Duncan W.J. (1937). Galerkin’s Method in Mechanics and Differential Equations.

[26] Van der Vorst H.A. (1992). Bi-CGSTAB: A Fast and Smoothly Converging Variant of Bi-CG for the Solution of Nonsymmetric Linear Systems. SIAM J. Sci. Stat. Comput..

[27] Schwarzenberg-Czerny A. (1995). On matrix factorization and efficient least squares solution. Astron. Astrophys. Suppl. Ser..

[28] Ba J.L., Kiros J.R., Hinton G.E. (2016). Layer Normalization. arXiv.

[29] Kingma D.P., Ba J. (2017). Adam: A Method for Stochastic Optimization. arXiv.

